# Ethical Principles in Medical Error Disclosure: From Harm to Healing in Clinical Practice

**DOI:** 10.21315/mjms-06-2025-493

**Published:** 2025-10-31

**Authors:** Yusrita Zolkefli

**Affiliations:** PAPRSB Institute of Health Sciences, Universiti Brunei Darussalam, Brunei Darussalam

**Keywords:** disclosure, ethics, trust, accountability, safety

## Abstract

The disclosure of medical errors constitutes a critical element of patient-centred care, one that is both ethically complex and indispensable. This paper explores how the principles of autonomy, beneficence, non-maleficence, and justice provide an ethical foundation for transparent communication, while also confronting challenges such as paternalism and systemic inequities that disproportionately affect marginalised communities. Institutional frameworks, such as the Communication and Optimal Resolution (CANDOR) process, are presented as mechanisms for advancing accountability, learning, and transparency. The discussion reinforces the need for ethical training, protective policies, psychological support for clinicians, and multidisciplinary collaboration. It further asserts that disclosure is a shared ethical responsibility, one that cultivates trust, promotes fairness, and contributes to the continuous improvement of safer and more equitable healthcare systems.

## Introduction

Medical errors have become a critical public health concern, having profound effects on patients, families, healthcare professionals, and healthcare systems (1). In addition to their clinical repercussions, these errors undermine the fundamental ethical principles of medical practice. The disclosure of medical errors has transitioned from a primarily legal obligation to a more comprehensive ethical obligation that is founded on the principles of public accountability, professional integrity, and patient autonomy (2). Honest and timely communication is indispensable for the preservation of trust, the promotion of collaborative decision-making, and the protection of patients’ rights to comprehensive and accurate information (3).

Despite this ethical clarity, numerous clinicians continue to be apprehensive about disclosing errors because of concerns regarding litigation, reputational damage, and punitive workplace cultures (4). This reluctance has the potential to exacerbate patient injury, undermine accountability, and erode public confidence in healthcare institutions (5). This paper examines the potential of the fundamental principles of biomedical ethics, autonomy, beneficence, non-maleficence, and justice, to regulate responsible and compassionate disclosure practices (6). It posits that effective and ethical communication necessitates not only individual moral commitment but also robust institutional support, ongoing professional training, and multidisciplinary collaboration.

## Ethical Principles and Challenges in Medical Error Disclosure

The fundamental biomedical ethical principles of justice, beneficence, autonomy, and non-maleficence underpin medical error disclosure. Autonomy necessitates that patients receive complete and accurate information, even in the event of errors (6). Patients are denied the capacity to make informed decisions, and trust in clinicians and institutions is undermined by the concealment of errors (7). Research indicates that patients place a high value on honesty and experience greater comfort when professionals disclose information transparently and candidly (8).

Healthcare professionals are obligated to prioritise patient well-being and prevent additional harm, as outlined in the principles of beneficence and non-maleficence. Despite concerns among some clinicians that disclosure may exacerbate patient distress, it is widely held that transparency often alleviates confusion and anxiety, thereby supporting emotional healing (9). The significance of maintaining a balance between honesty and sensitivity is underscored by national guidelines (10). Moreover, the “second victim” phenomenon, which involves clinicians experiencing emotional distress because of errors, has led to the creation of peer support initiatives that are designed to enhance the quality of disclosure and enhance resilience (11).

Fairness, accountability, and equity are required when patients are injured, as dictated by the principle of justice. This necessitates the immediate recognition of errors, the provision of candid explanations, and the implementation of suitable remedies (2). Globally, policies such as the United Kingdom’s Duty of Candour and apology legislation promote transparency and alleviate concerns regarding arbitrary punishment (12). By actively engaging patients and families in the development of policies and acknowledging their experiences, disclosure practices can develop into a more patient-centred, equitable, and responsive approach, which in turn fosters a greater sense of trust and collaboration in the healthcare sector (13).

Nevertheless, the implementation of these principles in clinical environments frequently results in intricate ethical dilemmas (6). For example, clinicians may withhold information to safeguard a patient’s psychological well-being, which may compromise autonomy, even if the motivation is non-maleficence. Although well-intentioned, this paternalism has the potential to undermine trust and impede transparency.

Additionally, these difficulties are further exacerbated by systemic disparities. How patients perceive and process information regarding medical errors is substantially influenced by factors such as age, cultural background, language proficiency, and socioeconomic status (14). Disparities in medication error reporting, for example, are disproportionately experienced by marginalised populations, both nationally and globally (15). These inconsistencies undermine justice by resulting in unequal outcomes and experiences, underscoring the necessity of disclosure policies that are patient-centred and culturally sensitive. While biomedical principles may not always provide straightforward solutions in complex and diverse clinical environments, they are an essential moral compass that guides deliberate and compassionate decision-making in the face of ethical challenges.

## Building Supportive Systems for Ethical and Effective Disclosure

To effectively address the ethical challenges associated with medical error disclosure, it is necessary to establish comprehensive institutional frameworks that prioritise transparency, emotional resilience, and ongoing learning, while simultaneously encouraging individual ethical responsibility.

The Communication and Optimal Resolution (CANDOR) process is a prime example of a structured approach to error disclosure that is consistent with both ethical and legal obligations. It follows eight critical steps: identifying the event, activating the CANDOR system, initiating timely disclosure to patients and families, investigating and analysing the event, achieving resolution, developing sustainable improvement plans, addressing scepticism and resistance, and recognising successes (16). Healthcare institutions cultivate a culture of safety, transparency, and open communication in response to adverse events by implementing CANDOR.

Integrating ethical principles into clinical practice through professional development is also essential for adequate disclosure. Simulation-based training and culturally sensitive communication seminars that are customised to diverse contexts can enhance clinicians’ confidence and proficiency in conducting respectful conversations about errors (17).

Next, institutions must establish explicit policies that require prompt, transparent disclosure and safeguard clinicians who report errors in good faith, thereby achieving a balance between accountability and compassion (13). Developing a culture of safety is crucial for reducing the stigma and dread associated with admitting mistakes. By modelling non-punitive responses and framing errors as opportunities for learning rather than punishment, leadership plays a pivotal role (18).

Meanwhile, recognising the “second victim” phenomenon and cultivating resilience, organisations should offer counselling and peer support programmes to assist healthcare professionals in their emotional well-being (19). Trust and respect are further fortified by engaging patients and families in continuous, candid dialogue (20).

**Figure 1:**
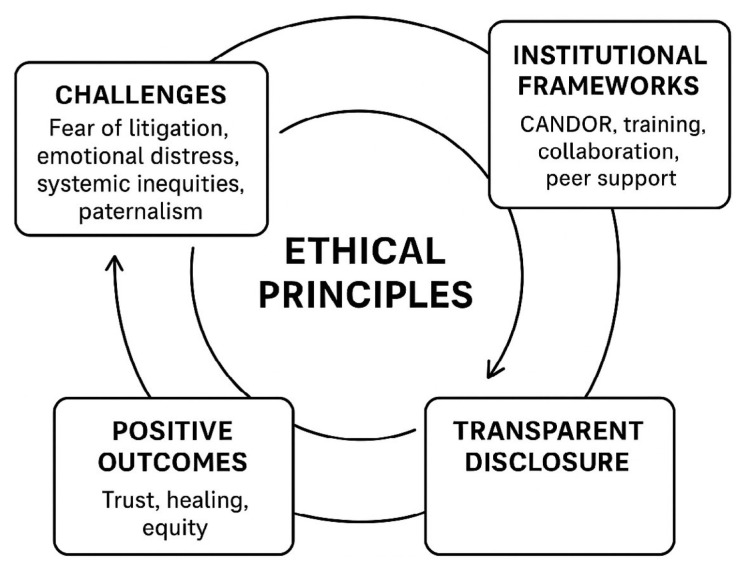
Ethics, transparency, and outcomes in medical error disclosure

A robust support system that attends to the ethical, legal, and emotional dimensions of disclosure is most effectively established through a multidisciplinary approach that incorporates the expertise of clinicians, legal advisers, ethicists, risk managers, and patient advocates (18). Lastly, accountability is reinforced, and a commitment to a culture of learning, honesty, and respect is demonstrated through continuous evaluation through patient feedback, incident analysis, and transparency in sharing findings (13). When these strategies are integrated, the process of error disclosure is reframed from a source of fear and contention into a constructive practice that promotes mutual trust, shared responsibility, and emotional healing. A framework linking ethics, transparency, and outcomes in medical error disclosure is illustrated in Figure 1.

## Conclusion

Preserving trust, integrity, and justice in healthcare necessitates the ethical obligation to disclose medical errors in complete transparency. One must undergo a systemic cultural transformation that prioritises accountability and learning to surmount obstacles such as fear of litigation and responsibility. Ethical leadership, explicit policies, and multidisciplinary collaboration, in addition to institutional frameworks such as CANDOR, are essential for supporting clinicians and promoting transparent communication. Recognising the emotional toll on healthcare professionals and offering the necessary assistance is essential for the maintenance of ethical disclosure. Incorporating these principles into healthcare training and organisational culture can transform errors from sources of harm into catalysts for meaningful improvement. This ultimately promotes equitable, patient-centred care and strengthens the ethical foundation of healthcare.
